# Health-Related Quality-of-Life after Laparoscopic Gastric Bypass Surgery with or Without Closure of the Mesenteric Defects: a Post-hoc Analysis of Data from a Randomized Clinical Trial

**DOI:** 10.1007/s11695-017-2798-z

**Published:** 2017-07-04

**Authors:** Erik Stenberg, Eva Szabo, Johan Ottosson, Anders Thorell, Ingmar Näslund

**Affiliations:** 1 Department of Surgery, Faculty of Medicine and Health, Örebro University, SE-701 85 Örebro, Sweden; 2 Department of Surgery, Ersta Hospital, Stockholm, Sweden; 3 Department of Clinical Sciences, Danderyd Hospital, Karolinska Institutet, Stockholm, Sweden

**Keywords:** Bariatric surgery, Laparoscopic gastric bypass, Small bowel obstruction, Internal hernia, Health-related quality-of-life, Randomized clinical trial

## Abstract

**Background:**

Mesenteric defect closure in laparoscopic gastric bypass surgery has been reported to reduce the risk for small bowel obstruction. Little is known, however, about the effect of mesenteric defect closure on patient-reported outcome. The aim of the present study was to see if mesenteric defect closure affects health-related quality-of-life (HRQoL) after laparoscopic gastric bypass.

**Methods:**

Patients operated at 12 centers for bariatric surgery participated in this randomized two-arm parallel study. During the operation, patients were randomized to closure of the mesenteric defects or non-closure. This study was a post-hoc analysis comparing HRQoL of the two groups before surgery, at 1 and 2 years after the operation. HRQoL was estimated using the short form 36 (SF-36-RAND) and the obesity problems (OP) scale.

**Results:**

Between May 1, 2010, and November 14, 2011, 2507 patients were included in the study and randomly assigned to mesenteric defect closure (*n* = 1259) or non-closure (*n* = 1248). In total, 1619 patients (64.6%) reported on their HRQoL at the 2-year follow-up. Mesenteric defect closure was associated with slightly higher rating of social functioning (87 ± 22.1 vs. 85 ± 24.2, *p* = 0.047) and role emotional (85 ± 31.5 vs. 82 ± 35.0, *p* = 0.027). No difference was seen on the OP scale (open defects 22 ± 24.8 vs. closed defects 20 ± 23.8, *p* = 0.125).

**Conclusion:**

When comparing mesenteric defect closure with non-closure, there is no clinically relevant difference in HRQoL after laparoscopic gastric bypass surgery.

## Introduction

It is well known that obese individuals report a lower health-related quality-of-life (HRQoL) than individuals with normal weight [1]. It is also widely accepted that bariatric surgery significantly improves HRQoL, mainly with regard to physical aspects [2–5]. The major contributing factor seems to be weight loss itself [4, 6]. Gastric bypass surgery is an efficient treatment for morbid obesity [7] and type II diabetes in obese subjects [8–10]. The introduction of the laparoscopic technique has improved the safety and morbidity related to the procedure [11, 12], and today, the method is generally considered to be safe [13]. Small bowel obstruction due to internal hernia, however, is considered to be one of the major downsides of laparoscopic gastric bypass surgery, with an incidence reaching 10% or higher if no preventive measures are undertaken [14–17]. Recently, closure of the mesenteric defects was shown to reduce the risk for small bowel obstruction, albeit at the price of a small increase in early small bowel obstruction due to kinking of the jejunojejunostomy [14]. However, complete evaluation of the efficacy of an intervention should also include patient-reported outcomes [18].

The aim of the present study was therefore to evaluate the effect of closure of the mesenteric defects during laparoscopic gastric bypass surgery on postoperative health-related quality-of-life.

## Methods

### Study Design and Participants

All patients fulfilling the criteria for bariatric surgery, as stated in the Swedish Board of Health and Welfare guidelines based on the NIH criteria [19], and operated during the inclusion period at any of the 12 participating centers were eligible for inclusion. Planned open procedures or conversion to open surgery before randomization were considered criteria for exclusion. Following intra-abdominal inspection at the beginning of the operation, the surgeon decided if the procedure could proceed laparoscopically. If that was the case, a sealed envelope was opened and the patient randomized to either mesenteric defect closure with running, non-absorbable sutures or non-closure. Patients were randomly assigned in a 1:1 ratio, with permuted blocks of different sizes, stratified by center. After the operation, randomization was open label.

### Data Collection

Data on baseline characteristics, operation data, and data from follow-up at 1 and 2 years were recorded in the Scandinavian Obesity Surgery Registry (SOReg) database. At baseline and at 1 and 2 years after surgery, all patients were asked to report on their health-related quality-of-life (HRQoL) using the short form 36 health survey (SF-36-RAND) [20] and the obesity-related problems (OP) scale [21].

The OP scale is a disease-specific scale measuring the impact of obesity on psychosocial functioning. The scale consists of eight questions on common obesity-related problems reported by bariatric surgery patients. The results are aggregated to form a total score ranging from 0 to 100, with low scores representing better well-being [21]. The SF-36 is a non-disease-specific survey consisting of eight dimensions of physical, social, and mental health with scores ranging from 0 to 100, with high scores representing better quality-of-life. In addition, the dimensions are summarized in a physical component summary (PCS) and a mental component summary (MCS), standardized to the general population, so that the mean score is 50 with a standard deviation of ten [21].

The difference in HRQoL between groups was analyzed as effect size (ES), calculated by the mean difference and divided by the pooled standard deviation [22]. The standard criteria of Cohen were used to define the magnitude of ES as trivial (0 to <0.2), small (0.2 to <0.5), moderate (0.5 to <0.8), or large (≥0.8) [4, 22].

### Outcomes

The main outcomes of the study were reoperation for small bowel obstruction and early severe postoperative complication [14]. HRQoL was measured and analyzed as a post-hoc analysis.

### Definitions

Comorbidity was defined as a condition requiring active pharmacological or continuous positive airway pressure treatment.

Reoperation for small bowel obstruction was defined as acute presentation of abdominal pain with signs of bowel obstruction at the time of surgery for this complication, i.e., dilatation of the small bowel/gastric remnant, mesenteric lymphedema, or incarcerated bowel.

### Statistics

Cox regression was used to compare reoperation rates for small bowel obstruction. Cumulative probability was estimated using the Kaplan-Meier technique. Categorical variables were analyzed with logistic regression. The *t* test was used to compare differences in health-related quality-of-life between the two groups. A *p* value <0.05 was considered to be statistically significant. All statistical analyses were made using SPSS Statistics version 22 (IBM Corporation, Armonk, NY, USA).

## Results

Between May 1, 2010, and November 14, 2011, 2587 patients were invited to participate in the study. Of these, 68 patients did not accept randomization. Ten patients were excluded due to conversion to open surgery before randomization or termination of the operation due to intra-abdominal pathology or for technical reasons. Further, two patients were excluded due to inclusion despite not meeting the inclusion criteria (planned for open surgery). The remaining 2507 patients were randomized to closure of the mesenteric defects (*n* = 1259) or non-closure (*n* = 1248). In the group randomized to closure of the mesenteric defects, 32 patients had neither of the mesenteric defects closed, 22 had only one mesenteric defect closed, and six were converted to open surgery after randomization took place. For unknown reason, five patients randomized to non-closure had the defects closed. The follow-up was on an intention-to-treat basis.

Follow-up at Day 30 was 99.8% (*n* = 2503), at 1 year 97% (*n* = 2439), and at 2 years 90% (*n* = 2245). With the addition of data from the Swedish national patient register, follow-up information on reoperation for small bowel obstruction at 2 years after surgery was 99% (*n* = 2482). At baseline, 1931 patients (77.0%) completed their HRQoL forms, of whom 47 patients (1.9%) had not answered the SF-36 and 11 patients (0.4%) the OP questions completely. At the 1-year follow-up, 1588 patients completed their HRQoL forms (63.3%), 24 patients (1.0%) had not answered the SF-36, and five patients (0.2%) the OP questions completely. At the 2-year follow-up, 1619 patients (64.6%) completed their HRQoL forms, 23 patients (0.9%) had not answered the SF-36, and one patient (0.0%) the OP questions completely.

There were no clinically relevant differences in baseline characteristics between the two groups (Table 1).

**Table 1:** Baseline characteristics

	Defects closed		Defects not closed	
		Missing data, *n*		Missing data, *n*
Gender		0		0
Male, *n* (%)	311 (25%)		333 (27%)	
Female	948 (75%)		915 (73%)	
Age, (mean ± SD), years	41.7 ± 10.8	0	41.7 ± 10.7	0
Comorbidity, *n* (%)	618 (49%)	0	636 (51%)	0
Sleep apnea, *n* (%)	107 (8%)	0	100 (8%)	0
Hypertension, *n* (%)	327 (26%)	0	333 (27%)	0
Diabetes, *n* (%)	179 (14%)	0	146 (12%)	0
Dyslipidemia, *n* (%)	139 (11%)	0	150 (12%)	0
Dyspepsia/GERD, *n* (%)	117 (9%)	0	127 (10%)	0
Depression, *n* (%)	175 (14%)	0	174 (14%)	0
Other disorders, *n* (%)	83 (7%)^a^	0	77 (6%)^b^	0
Previous venous thromboembolism, *n* (%)	39 (3%)	251	33 (3%)	266
Smoking, *n* (%)		193		178
Active smoking	174 (14%)		176 (14%)	
Previous history of smoking	160 (13%)		179 (14%)	
Body mass index, (mean ± SD), kg/m^2^	42.3 ± 4.9	0	42.4 ± 5.2	0
Waist circumference, (mean ± SD), cm	127.5 ± 13.1	115	127.2 ± 13.4	124

^a^Other disorders were (in percentage of all patients randomized to mesenteric defect closure) cardiovascular disease 0.6%, pulmonary disease 0.5%, pain or mobility limitation 3.8%, systemic disease 0.6%, psychiatric disorder other than affective disorder 0.1%, and other 0.2%

^b^Other disorders were (in percentage of all patients randomized to non-closure) cardiovascular disease 1.0%, pulmonary disease 0.4%, pain or mobility limitation 4.4%, systemic disease 0.4%, psychiatric disorder other than affective disorder 0.2%, and other 0.1%

By 2 years after surgery, 78 patients (cumulative probability 6.4%) with non-closure of the mesenteric defects and 51 patients (cumulative probability 4.1%) with closed mesenteric defects had been reoperated for small bowel obstruction (*p* = 0.013).

### Health-Related Quality-of-Life

**Fig. 1:**
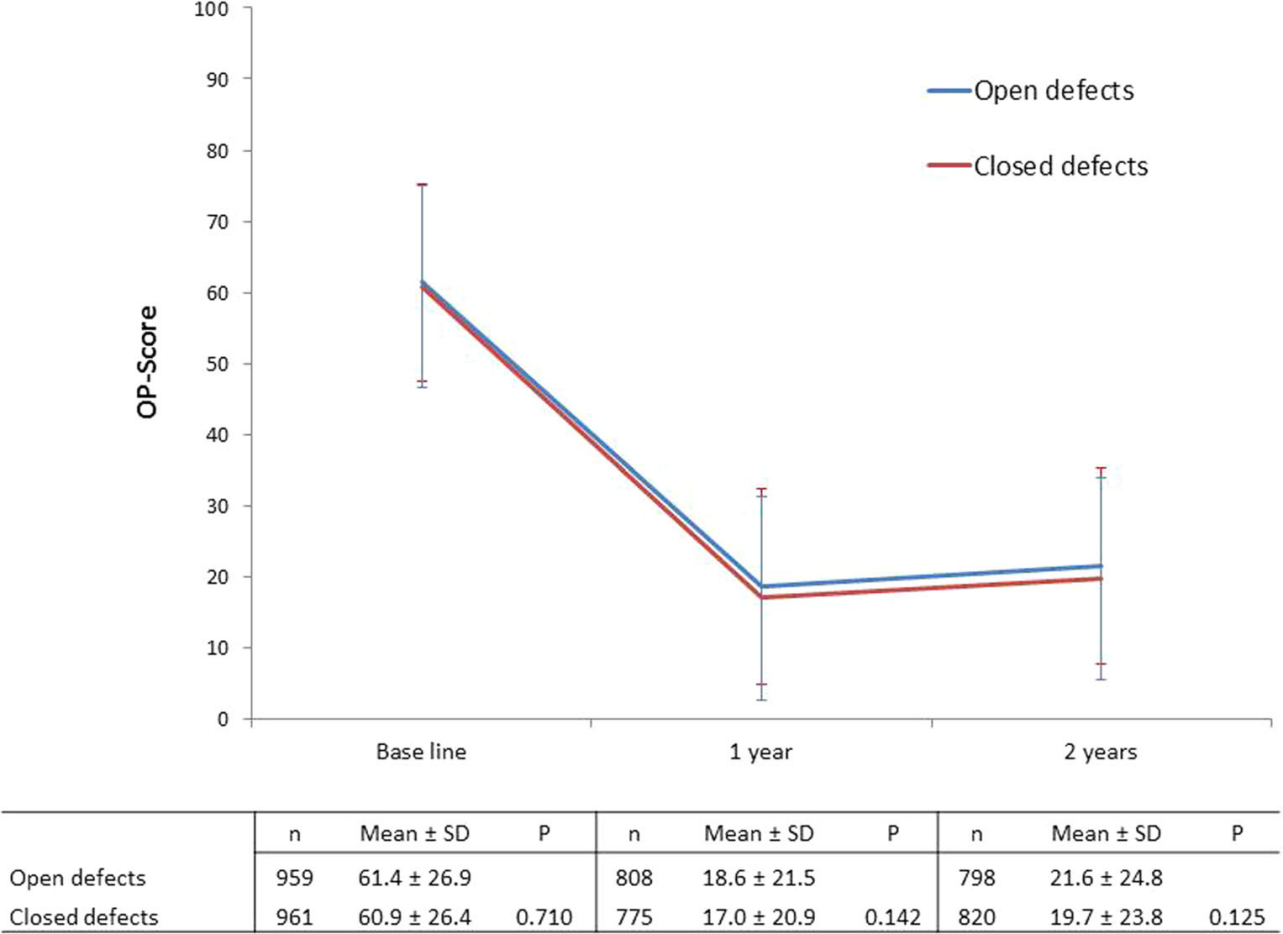
OP score for the two study groups at baseline, 1 and 2 years after the operation

At 1 and 2 years after surgery, HRQoL on the OP scale had improved in both groups (Fig. 1). Improvements in physical aspects of HRQoL (Table 2) and specific dimensions of mental HRQoL (Table 3) on the SF-36 were also observed. Patients with closed mesenteric defects experienced improvement in MCS at 1 and 2 years postoperatively, whereas no significant difference was seen for patients with open defects.

**Table 2:** HRQoL SF-36 physical scores

	No.	PSC		PF		RP		BP		GH	
		Mean ± SD	*P*	Mean ± SD	*P*	Mean ± SD	*P*	Mean ± SD	*P*	Mean ± SD	*P*
Baseline											
Open defects	942	39 ± 10.8		63 ± 21.3		61 ± 39.3		58 ± 26.9		61 ± 21.3	
Closed defects	942	39 ± 10.5	0.700	63 ± 21.9	0.442	63 ± 38.4	0.346	58 ± 27.1	0.932	61 ± 21.4	0.954
1-year follow-up											
Open defects	799	51 ± 8.7		90 ± 15.2		87 ± 28.8		75 ± 28.1		80 ± 20.0	
Closed defects	765	51 ± 8.5	0.782	90 ± 15.8	0.995	89 ± 27.6	0.292	76 ± 27.6	0.451	81 ± 19.2	0.529
2-year follow-up											
Open defects	787	51 ± 9.5		90 ± 16.4		85 ± 32.3		72 ± 36.3		76 ± 22.6	
Closed defects	809	51 ± 9.7	0.599	89 ± 17.8	0.751	86 ± 30.2	0.268	74 ± 28.6	0.113	77 ± 21.9	0.187

*PCS* physical health component summary score, *PF* physical functioning, *RP* role-physical, *BP* bodily pain, *GH* general health

**Table 3:** HRQoL SF-36 mental scores

	No.	MCS		VT		SF		RE		MH	
		Mean ± SD	*P*	Mean ± SD	*P*	Mean ± SD	*P*	Mean ± SD	*P*	Mean ± SD	*P*
Baseline											
Open defects	942	48 ± 11.3		49 ± 23.0		76 ± 25.4		78 ± 35.6		73 ± 18.4	
Closed defects	942	47 ± 11.5	0.225	48 ± 24.3	0.365	75 ± 26.3	0.476	76 ± 36.5	0.173	73 ± 18.9	0.621
1-year follow-up											
Open defects	799	50 ± 11.5		69 ± 23.5		88 ± 21.0		87 ± 29.9		81 ± 20.0	
Closed defects	765	50 ± 10.5	0.149	70 ± 22.7	0.479	90 ± 19.1	0.067	88 ± 28.7	0.520	83 ± 17.6	0.088
2-year follow-up											
Open defects	787	47 ± 13.0		64 ± 25.5		85 ± 24.2		82 ± 35.1		77 ± 21.4	
Closed defects	809	48 ± 12.2	0.114	64 ± 25.4	0.596	87 ± 22.1	0.047	85 ± 31.4	0.027	78 ± 20.9	0.489

*MCS* mental-health component summary score, *VT* vitality, *SF* social functioning, *RE* role-emotional, *MH* mental health

HRQoL was similar in both groups at baseline (Table 2 and Fig. 1). No significant differences were seen between the two groups on the OP scale (Fig. 1). In the SF-36, patients with closed mesenteric defects reported slightly better HRQoLs with regard to social functioning (ES = 0.1) and role emotional at the 2-year follow-up (ES = 0.1; Table 2).

Irrespective of closure of the mesenteric defects or not, patients reoperated for small bowel obstruction reported lower HRQoL for some aspects of physical and mental HRQoL when compared with patients not experiencing this complication. At the 2-year follow-up, differences were seen on the SF-36 regarding physical role (74 ± 41.4 vs. 86 ± 30.5, *p* = 0.013, ES = 0.3), general health (71 ± 23.1 vs. 77 ± 22.2, *p* = 0.019, ES = 0.3), vitality (54 ± 26.1 vs. 64 ± 25.3, *p* = 0.001, ES = 0.4), and social functioning (79 ± 28.3 vs. 86 ± 22.9, *p* = 0.046, ES = 0.3). No significant difference was seen on the OP scale (SBO 21 ± 24.3 vs. no-SBO 23 ± 23.3, *p* = 0.398).

When patients reoperated for SBO were excluded from the analysis, no difference in HRQoL was seen on the OP scale between patients with or without closure of the mesenteric defects (open defects 22 ± 24.9 vs. closed defects 20 ± 23.8, *p* = 0.093). On the SF-36, patients randomized to mesenteric defect closure still reported slightly higher scores for social functioning (open defects 85 ± 24.2 vs. closed defects 87 ± 22.1, *p* = 0.041, ES = 0.1) and role emotional (open defects 82 ± 35.0 vs. closed defects 85 ± 31.5, *p* = 0.031, ES = 0.1).

Patients who failed to report on HRQoL at 2 years were younger (40.1 ± 10.28 years vs. 42.7 ± 10.88, *p* < 0.0001), had a higher preoperative BMI (42.8 ± 5.37, vs. 42.1 ± 4.84, *p* = 0.001), and were more often active or previous smokers (35.9% vs. 30.3%, *p* = 0.008), but there were fewer with sleep apnea (6.1% vs. 9.5%, *p* = 0.003), hypertension (22.5% vs. 28.4%, *p* = 0.001), and dyslipidemia (9.8% vs. 12.5%, *p* = 0.045).

## Discussion

In the present study, HRQoL was significantly improved after gastric bypass regardless of mesenteric defect closure or not. As reported previously [2, 3, 5, 23, 24], HRQoL was particularly improved in aspects related to obesity-related problems and physical aspects of HRQoL, while a much smaller effect was seen on mental aspects of HRQoL. Closure of the mesenteric defects was associated with a slightly better improvement in some aspects of HRQoL compared to non-closure, and these differences remained even when patients reoperated for small bowel obstruction were excluded from the analysis. The instruments used to measure HRQoL, however, are very sensitive in discovering small differences, particularly when being used on large study-cohorts. Based on ES, the small differences seen between the two groups in this study cannot be regarded as clinically relevant [4, 22].

Closure of the mesenteric defects is rapidly gaining acceptance as an efficient way to reduce the risk for small bowel obstruction after laparoscopic gastric bypass surgery [14]. The efficacy of this intervention, however, must also be evaluated in the light of complications associated with closure itself, as well as patient-reported experiences. In this respect, the technique has been shown to be associated with a small but significant risk for kinking of the jejunojejunostomy resulting in small bowel obstruction in the early postoperative period [14, 25]. Moreover, gastric bypass itself is also known to be associated with development of chronic abdominal pain in some patients, symptoms that are known to reduce HRQoL after bariatric surgery [5, 26, 27]. One concern that has been raised concerning mesenteric defects closure is that this intervention might contribute to the development of chronic abdominal pain, which in turn would probably affect HRQoL [18].

To our knowledge, the present study is the first to compare HRQoL in patients after laparoscopic gastric bypass surgery with or without mesenteric defect closure. Despite a slightly better improvement in some aspects of HRQoL, our results indicate that mesenteric defect closure during the primary operation does not affect HRQoL after surgery. Consequently, from a quality-of-life perspective, our results do not contradict that the mesenteric defects should be closed during laparoscopic gastric bypass surgery [14]. Patients who suffered from small bowel obstruction within the first 2 years after surgery reported a lower HRQoL according to the SF-36 survey. Although the ES of these differences were small, they are important to bear in mind. Patients who suffer from more severe postoperative complications as well as patients with complications of more chronic character report a lower HRQoL after surgery [5, 26–28]. For many patients, deterioration in quality-of-life is one of the most important factors bringing them to surgery. Suffering a postoperative complication, particularly if this affects HRQoL, reduces the success of the operation. Small bowel obstruction, particularly when caused by internal herniation, is a potentially very serious complication of laparoscopic gastric bypass surgery, associated with severe morbidity as well as mortality. Furthermore, as suggested by the results of the present study, these patients experience a reduction in their HRQoL. Closure of the mesenteric defects is therefore an important measure to reduce the risk for this complication.

One limitation of this study is that one in every three patients did not report on their HRQoL. However, this follow-up rate is comparable with that of other studies focusing primarily on patient-reported outcome [2, 5, 27]. Patients who failed to report on their HRQoL were younger, had a slightly higher BMI, and more often had a history of smoking, but less often had comorbidity. Younger age and smoking are factors known to be associated with both pain and chronic symptoms after gastric bypass surgery [27, 29]. The loss to follow-up in our study may therefore result in a slight overestimation of the effect of closure of the mesenteric defects on HRQoL, although it is unlikely that this would impose a differential bias on the results since the missing data were equally distributed between the groups. Furthermore, the study covers the first 2 years after surgery only. This is the time when patients have lost most weight and generally rate their HRQoL highest [4, 30]. The known complications of mesenteric defect closure, however, mostly occur in the early postoperative phase whereas the benefits come with time [14, 25]. It thus seems unlikely that patients with mesenteric defect closure would report a greater reduction in HRQoL than patients with no closure once the 2-year mark has passed. Finally, HRQoL was estimated using two validated scales. A full evaluation of patient’s experience of mesenteric defect closure will need further studies with a more qualitative approach.

In conclusion, the results of this study do not invalidate previous recommendation that the mesenteric defects should be routinely closed during laparoscopic gastric bypass procedures [14].
